# A model-driven PBL application to support the authoring, delivery, and execution of PBL processes

**DOI:** 10.1186/s41039-016-0030-8

**Published:** 2016-02-25

**Authors:** Disi Wang, Mohammed Samaka, Yongwu Miao, Zeyad Ali, H. Ulrich Hoppe

**Affiliations:** 1 Department of Computer Science and Applied Cognitive Science, University of Duisburg-Essen, Lotharstr. 63, 47057 Duisburg, Germany; 2 Computer Science and Engineering Department, Qatar University, Doha, Qatar

**Keywords:** PBL, Learning design, Graphical authoring tool, Model-driven architecture (MDA), PBL script, Semi-structured data, Data management, Web-based application

## Abstract

As problem-based learning (PBL) is becoming more and more popular, there is also a growing interest in developing and using technologies in the implementation of PBL. However, teachers may have difficulties to design and deliver a pedagogically well-designed and technically smoothly executable online or blended PBL process on their own because they lack appropriate expertise in learning theories and design methods as well as a deeper understanding of the potential affordances of the available technologies. From this premise, we are committed to developing and testing methods and tools to support the design and delivery of online or hybrid PBL processes with high flexibility and a low threshold of usage requirements. This paper presents a technical approach to develop a web-based PBL application that supports both authoring and run-time usage. In comparison with other tools and technical approaches, it is concluded that a combined use of a model-driven approach and semi-structured data management appears to be a promising approach to effectively and efficiently support the authoring, delivering, and execution of design-time and run-time PBL processes.

## Introduction

Problem-based learning (PBL) is a learning method to structure learning activities in such a way as to confront students with problems from practice as a stimulus for learning (Boud and Feletti 1998). It engages students in an active, collaborative, student-centered learning process (or a series of learning steps) that develops critical thinking, problem-solving, teamwork, and self-directed learning abilities needed to meet the challenges of life and career in our increasingly complex environment (Hmelo-Silver and Eberbach 2012). PBL has been successfully used in various different domains, and its benefits have been largely demonstrated (Savery 2006). The application of new technologies such as Web 2.0 and virtual collaboration environments can enrich and improve implementations of PBL (Kaldoudi et al. 2008). Nevertheless, to achieve its full power, an online or blended PBL process needs to be well designed, and a sound online or blended PBL process may be a collaborative product of years of research, application, assessment, and redesign. Usually, designing such a PBL process is an intensive mental work involving implicit decisions. Traditionally, the teacher individually constructs a PBL design based on personal experience and represents it in natural language as a course or lesson plan or as a learning scenario on paper. Teachers may have neither appropriate expertise in learning theories and design methods nor a deep understanding of the potential affordances of technologies. They lack guidance to design a high-quality and technology-supported PBL plan in a specific context in order to benefit from PBL and new technologies.

The work described in this paper has been developed in the context of the PLATE (Problem-based Learning Authoring and Transformation Environment) project. PLATE aims at facilitating teachers in the design, representation, understanding, communication, customization, reuse, transformation, and execution of online or blended PBL processes in an effective, efficient, and flexible manner. We try to help teachers to make the implicit design process explicit in order to improve design quality and to represent a traditionally informal description as a formal model that can be used for scaffolding and orchestrating a PBL process. Various challenges need to be overcome to make this vision come true. One major challenge is to find an appropriate way to apply the research achievements in the area of PBL pedagogy to the contemporary information and communication technology (ICT). Therefore, this paper intends to present our technical approach as a fundamental support for the PBL implementation. For this purpose, we developed a web-based PBL application. This integrated application consists of a PBL authoring tool, a PBL script instantiation tool, and a PBL-specific run-time environment. We claim that applying a model-driven approach with a semi-structured data management can make up an effective combination to technically support the authoring, delivering, and execution of the design-time and run-time information of PBL processes.

The rest of the paper is organized as follows: The “Characterize PBL and identify technical requirements” section characterizes the design and the implementation of a PBL process as a basis for creating a new sort of PBL application. “The PBL application” section presents the functionalities of our application to provide a basic impression about how the application empowers teachers in PBL process design and technical implementation. The “A model-driven approach” section illustrates the underlying model-driven approach. Here, we can see how a PBL process is transformed from a lower level abstract model to a higher level executable model for process design and technology-enhanced implementation. The “Supporting the manipulation of PBL processes under the model-driven approach” section presents a semi-structured data management method based on the model-driven approach in order to effectively represent, manage, and deliver flexibly structured PBL processes. The “Related work” section compares this application with related work. The final section summarizes our work and describes the future work.

## Characterize PBL and identify technical requirements

PBL can be conducted in a number of ways based on different models such as the McMaster PBL model (Woods 1996), the Maastricht “seven-jump” model (Barrows 1996), the Aalborg “problem oriented project pedagogy (POPP)” model (Dirckinck-Holmfeld 2002), Seymour’s “five-stage” model (Seymour 2010), and the Salford model (McLoughlin and Davrill 2007). In order to illustrate how a PBL process is usually described informally in natural language, we take the seven-jump model description from Maurer and Neuhold (2012) as an example through this paper:

To get students started on a certain topic, they are confronted with an assignment that … outlining the problem or asking for a specific task to complete. … Students are supposed to have read and looked at this assignment already before their tutorial (or during the break), so that they can start with **clarifying terms and concepts**. This first step guides students mentally into the topic, and by discussing unknown words or concepts it is ensured that all students understand the text as it stands and that the group shares ideas about illustrations that might be part of the assignment. This first step provides a common starting point and leads the group into the topic. In the next step, the whole group agrees on the **formulation of the problem statement** that frames the whole assignment, provides a title for the session, and makes the group agree on what the general impetus of the assignment is about. Problem statements can take the form of more traditional titles, but are sometimes also formulated as broader research questions or provoking statements.

The problem statement should trigger the next step of the **brainstorm**. … Everything is allowed during this step, and ideas are collected unquestioned at the whiteboard (i.e. there are no wrong ideas; everyone should be allowed to follow her/his own ideas). … The outcome of the brainstorm is noted on the whiteboard by the secretary that during the next (fourth) step should be **categorized and structured** by the students. … but by structuring the brainstorm students categorize keywords that fit together and in this way they find common patterns that in the next step will allow for the formulation of specific questions. As last step of the pre-discussion, students agree on the **formulation of common learning objectives**, by referring to the brainstorm and the now structured collection of ideas that they have noted on the whiteboard. …

After these five steps of the pre-discussion, students leave the group again to engage in the **self-study**, which takes a central position in the PBL framework and emphasises the self responsibility of the learner for knowledge acquisition. During this self-study students should work on their individual answers to the formulated learning objectives. … The following tutorial, normally taking place two or three working days later, starts with the **post-discussion** where students report back, exchange their answers, discuss problems and try to come to common conclusions of how to answer the learning objectives. … By experiencing different perceptions of a question by their peers, … students are acquainted to report, listen, discuss and debate.

As the name implies, we can see from the citation that this PBL model consists of seven steps which include *clarifying terms and concepts*, *formulation of the problem statement*, *idea brainstorming*, *categorizing and structuring ideas*, *formulation of learning objectives*, *resolution through self-study*, and *conclusion by peer evaluation*. Looking into the first step, we can find that it consists of three activities: *reading assignment*, *discussing unknown words or concepts*, and *understanding the text as it stands*. In fact, in each step, one or several activities will be performed by the facilitator, individual students, student groups, or other stakeholders, e.g., scientific staff. In practice, one needs to extend or modify the model to fit the specific learning context. In particular, how these steps and activities will be actually arranged depends on concrete situations such as the number of students, the learners’ prior knowledge and PBL skills, the group structures, the problem used to drive the learning, the topics to be learned, and the availability of learning technologies.

In order to characterize the PBL process and identify technical requirements to support online or hybrid PBL processes, we have worked out a scenario named “deformed frogs” according to the seven-jump model. In this PBL scenario, we assume that the PBL process is conducted in a classroom with a digital whiteboard at the front and a PC for each student. The students involved in this scenario are divided into several small groups. The learning process starts with a facilitator giving reading assignments and materials about the discovery of deformed frogs in a local area to students. This challenges the students to investigate the status of the frog population and encourage them to take a proactive stand on this environmental concern. Then, the facilitator coaches the students to identify and understand the problem. After a discussion, the students identify the problem: “what is the cause of deformity of the frog and how to prevent it from spreading?” The students identify major issues connected to the problem. The identified issues were frog habitat, the various types of deformities in frogs, wetlands, watersheds, the effects of pollution on a natural habitat, and so on. In the scenario, the students acquire knowledge through presenting, arguing, and evaluating the hypotheses and solutions.

**Fig. 1:**
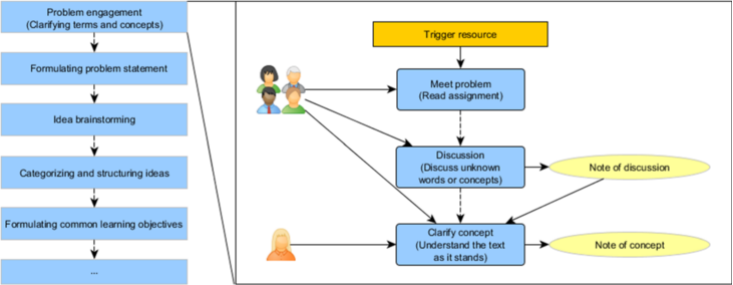
The process structure of the PBL scenario deformed frogs

Figure 1 depicts the structure of the PBL scenario described above. The left part of the diagram presents a sequence of steps as the overall process of the scenario. We call each step as a PBL phase. The right part of the diagram illustrates the activity structure of the first phase. In this phase, students in the class meet the problem by reading assigned learning materials at first. Then, they discuss the unknown words or concepts in groups. Each group creates a list of unknown words or concepts. Finally, the facilitator helps students to clarify the unknown words or concepts and to produce notes as the learning outcome of the phase. All these activities are carried out by using the digital whiteboard. Some activities produce artifacts or need learning resources.

Some important PBL research so far tried to support this kind of learning process by applying information and communication technologies (Kaldoudi et al. 2008). However, there is still a deficit in flexible and powerful utilization of ICT. On the one hand, some of the present ICT applications for PBL lack flexibility to support different models. For example, STELLAR (Hmelo-Silver et al. 2009) supports conducting the PBL process through a nine-step model; ePBL (Ali and Samaka 2013) is based on the McMaster PBL model. Although these applications are easy to use and have a well-designed PBL process pattern inside, they are rigid, which means teachers cannot configure the sequence of learning activities or customize certain activity units for their particular practices or purposes. For example, it would be difficult to implement our deformed frogs scenario in these approaches, or some other teachers wanted to apply Seymour’s five-stage model that would be impossible. On the other hand, some applications, such as LAMS (Dalziel 2003), are flexible enough but inadequate to help teachers in conducting sound PBL processes, especially when teachers do not have enough understanding of PBL pedagogy. When designing a PBL process, teachers need to figure out which activities are appropriate for which phase or what kinds of artifacts should be provided as temporary or final learning outcomes for certain phases. As a result, for the current PBL implementation, the process design is usually just embedded in teachers’ practice, and the process design ideas tend to be implicit. This leads to that PBL processes are mostly implemented only based on the social protocol and the manual configuration of various application tools, as well as the manual management of learning resources and (non-) digital learning artifacts.

Faced with these facts, it is necessary to find a new more flexible way of technically supporting and empowering teachers in implementing PBL. To achieve this, we consider that the following requirements should be met: First, the technical support should be able to help teachers, who may be not fully familiar with PBL and may not have comprehensive technical knowledge to technically represent their PBL ideas. Second, it should be possible that PBL processes can be created based on different PBL models and can be flexibly customized and applied to different learning contexts. Third, it is required to automatically scaffold and orchestrate the processes for facilitator and learners in an online or blended learning manner to some extent.

To meet all the requirements described above, we have developed a flexible, web-based PBL-specific application in PLATE. Being able to support those requirements ensures the application to be able to provide a balance point of the flexibility and easy-to-use responsibility, which could accordingly help teachers move from their traditional teaching methods to the ICT-enhanced PBL more smoothly. The next section will directly illustrate in detail the major functionalities being implemented in the application through carrying out the example scenario deformed frogs. The reasons that we first show our implementation result are (1) to make readers have an intuitive understanding how the application meet the requirements and (2) to depict a more comprehensive but clear picture which will help us to explain why there is the necessity of adopting the model-driven approach and developing a semi-structured data management method in an integrated manner to make the application implementation possible.

## The PBL application

**Fig. 2:**
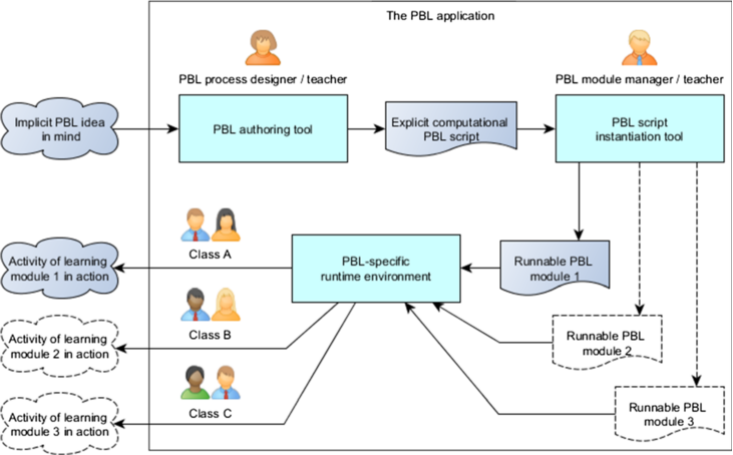
The major functional modules of the PBL application

Our PBL application consists of three major functional modules: a *PBL authoring tool*, a *PBL script instantiation tool*, and a PBL-specific *run-time environment*. Figure 2 depicts the relationships between the three modules and their target users. Before presenting the different specific functionalities, it is worth clarifying that in this paper, we assume the users of the *authoring tool* to be PBL teachers, users of the *instantiation tool* to be PBL (process) designers, and users of the *run-time environment* (process run-time) to be facilitators.

As shown in Fig. 2, a PBL designer represents his or her implicit PBL design ideas using the authoring tool to gradually create an explicit and formal PBL process under the guidance support of the system. The process is stored in the application as a PBL script. The designer then instantiates the PBL script through using the instantiation tool in order to make the PBL script runnable as different PBL modules for different classes. The runnable modules demonstrate the different concrete situations such as the different groupings of students or different learners inside groups or different learning resources. Finally, the run-time environment handles all the PBL modules adaptively so that learners in their classes are presented with their corresponding deliverables, resources, and tools since all the activities are timely structured and assigned according to the instantiated concrete situation and the design ideas embedded in the learning process.

Figure 2 shows the conceptual view of the entire application. The following sub-sections demonstrate the functionalities of each module in details.

### The PBL authoring tool

**Fig. 3:**
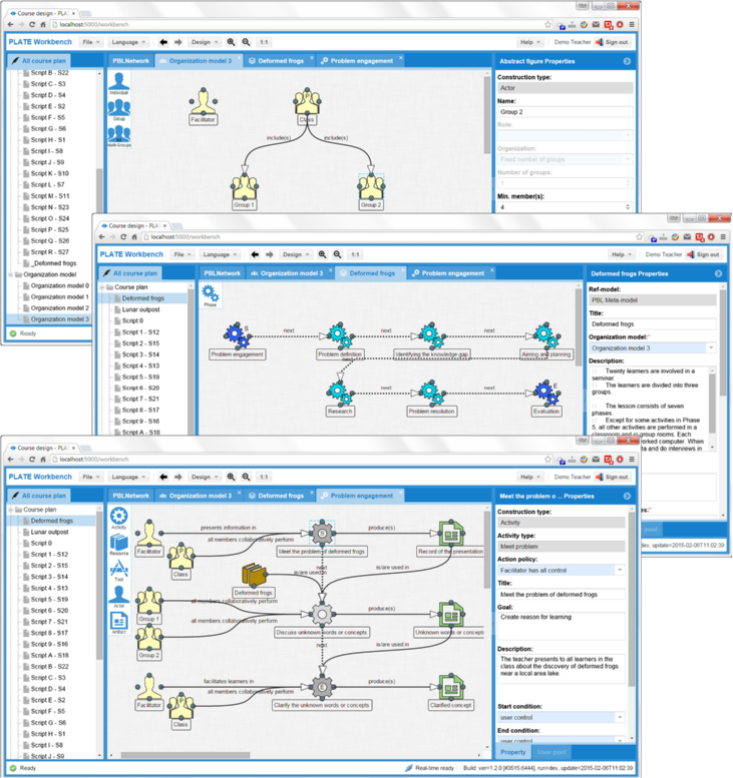
User interfaces of the actor organization editor and the PBL phase-activity process editor in the PBL authoring tool

Figure 3 shows the typical user interfaces (UIs) of the PBL authoring tool when presenting the deformed frogs learning scenario. As the most important module of the application, the authoring tool consists of two authoring editors: an *actor organization editor* and a *phase-activity process editor* (simply *process editor*). In this figure, from top to bottom, the three screen-shots correspondingly indicate the organization modeling in the actor organization editor and the phase process authoring and the activity process authoring in the phase-activity process editor.

All of the three UIs have similar components with operation-alike functionalities to provide a higher usability. The middle of each screen-shot is a graphical workspace in which the designer authors and structures the organizational model or the phase-activity process. The graphical workspace has a dynamic list of building blocks at the top-left corner of each graphical workspace. The dynamic building blocks are context appropriate for building the actor organization or the phase-activity process. On the left of the workspace, a process-script file management panel is provided for handling the created process scripts for each PBL designer. On the right, there is a context-aware property editing panel that is used to show and set the properties of the selected actor or phase-activity element in the workspace.

When modeling the actor organization through the *organization editor*, the designer can simply drag the “actor” icon from the building block list and drop it onto the graphical workspace to create an actor. The application will tell the designer that the type of actor could be *individual*, *group*, or *multiple groups*. Theoretically, these three types of actors are sufficient to make the designer build a complex enough organization model. As shown in the first screen-shot, we build a very simple class organization model. In this model, an individual actor is created and named as “facilitator”; a group actor is created and named as “class.” Two sub-groups, “group 1” and “group 2,” are added underneath the class. In the property panel, we can see that group 2 is specified to have a maximum of four participants. The designer then creates directed connections between the class and the groups. The connection between actors is important in defining the element relationship for the organization. With the connections, the class becomes a parent group of both group 1 and group 2.

Similar steps are performed when designing phase-activity processes through the process editor. The second screen-shot in Fig. 3 shows a PBL phase-activity process which is made up of several phase elements with several directed connections indicating the process sequence. The third screen-shot shows the internal process structure of an upper level phase element. Here, the three ordered activity elements are associated with other types of elements such as actors and artifacts. In comparison with the actor organization editor, there are more element types such as *phase*, *activity*, *resource*, *tool*, *actor*, and *artifact*, while only the actor element type is available in the actor organization editor.

Generally speaking, the process editor scaffolds the phase-activity process design from two perspectives. First, at the moment a designer puts a phase element onto the workspace, a pop-up window will list all available phase types. These types come from different models. For example, from the type list, the designer can find the phase type such as *clarifying terms and concepts* or *formulation of the problem statement* to create a seven-jump model-based phase, or he or she can also choose the phase type such as *dependency and inclusion* or *counter-dependency and fight* to create a five-stage model-based phase. Then, when creating activity elements under a created phase, operations invalid or inappropriate to the current phase context are blocked. As shown in Fig. 3, the second screen-shot represents the seven learning phases in order to describe the whole learning process of the deformed frogs in a higher abstraction level. Then, each phase can be opened like a folder to go to the lower abstraction level, which is shown by the third screen-shot. In the figure, the third screen-shot depicts the detailed activity process (represented by the gray gears) of the first phase “problem engagement” (created by choosing the phase type clarifying terms and concepts). In this level of the authoring, the designer is guided to create activities only with the activity types such as *meet problem*, *clarify concept*, and *discuss* under the context of the phase type clarifying terms and concepts. The same concept is applied for creating artifacts; options like *record* and *clarify concept* are found in the artifact-type list.

Actors in the learning process are the actors created in the actor organization editor. They can be assigned to activities as shown in the figure. *Engagement mode*s can be set between actors and activities, where the engagement mode is the nature of the roles that the participants are expected or required to play while they are performing a learning task on the activity. For instance, when the designer assigns the class to the activity “meet the problem of deformed frogs,” as shown in the third screen-shot, he or she is guided to select one engagement mode such as *each member individually performs*, *all members collaboratively perform*, and *each group separately performs*. As we can see in the screen-shot, the all members collaboratively perform is chosen.

**Fig. 4:**
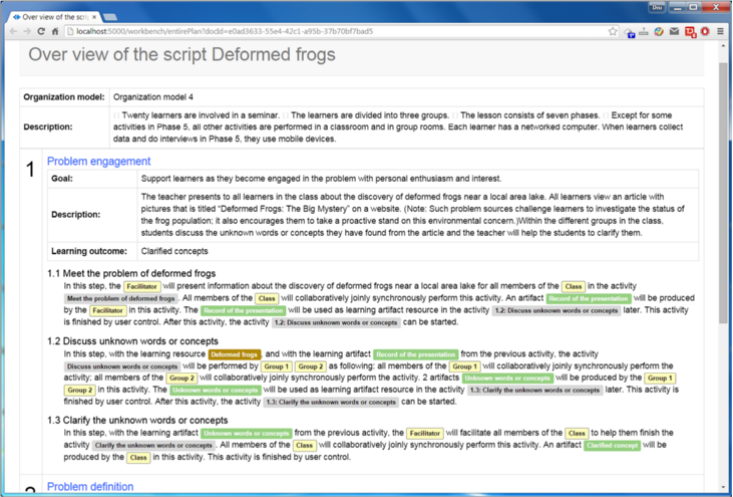
The textual output of the graphical represented PBL process

The graphical representation of a PBL process will be handled as a single PBL script by the tool. The script is stored in a PBL script repository for the purpose of retrieving, sharing, and reusing, as well as can be exported as a textual script file. The textual script exporting provides an alternative way that could enable the designer to review or share his or her design entirely as well as to let learning participants have an overview of the whole learning process. Figure 4 shows the textual script of the example deformed frogs. Notice that if the designer did not specify the “goal” or “description,” the tool will automatically generate a predefined text corresponding to the element type.

In this textual script, the top level contains the phases; then, each phase is detailed with its own activities. In phase 1 problem engagement, we can see each activity has an automatically generated summary according to the actors, resources, tools, and artifacts involved with it. For example, the generated summary description of activity 1.2 “Discuss unknown words or concepts” is

In this step, with the learning resource *Deformed frogs*, and with the learning artifact *Record of the presentation* from the previous activity, the activity *Discuss unknown words or concepts* will be performed by *Group 1 Group 2* as following: all members of the *Group 1* will collaboratively jointly synchronously perform the activity; all members of the *Group 2* will collaboratively jointly synchronously perform the activity. 2 artifacts *Unknown words or concepts* will be produced by the *Group 1 Group 2* in this activity. The *Unknown words or concepts* will be used as learning artifact resource in the activity 1.3: *Clarify the unknown words or concepts* later. This activity is finished by user control. After this activity, the activity 1.3: *Clarify the unknown words or concepts* can be started.

This functionality shows that the tool has the capability of comprehending the meaning of the graphical process representation and can help designers to transform their in-mind design idea into a computer- and also human-understandable script. The tool is also designed to be able to translate the script into other scripts that can be run in other run-time learning applications, for example, the IMS Learning Design (IMS-LD)-compatible players.

### The instantiation tool

Through providing a graphical UI to the formal representation, the PBL authoring tool supports PBL designers to express their PBL processes relatively easily. The graphical representations are stored as PBL scripts in the application. However, a script is actually just an abstract process model that does not come with concrete realizations. In other words, there are no particular learners or facilitators linked to the activities of the process, and the timely structure of the activities is yet unavailable. This means that the process is still not in a runnable state at this stage; it needs to be instantiated. For this purpose, an instantiation tool has been specifically developed, in order to support the management of the PBL modules. In this paper, we refer to an instantiated PBL process as a PBL module.

**Fig. 5:**
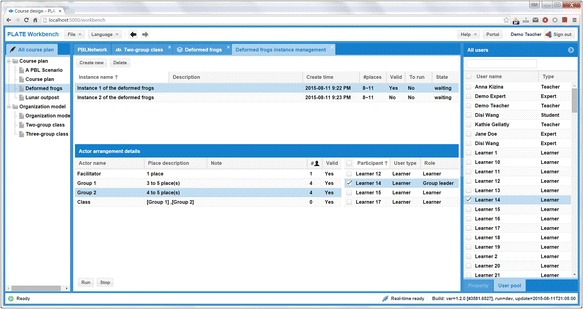
The UI of the script instantiation tool

The layout of the instantiation tool basically consists of three areas as shown in Fig. 5. The left-side panel lists all the previously generated process scripts. The middle part reflects the instantiation details of the selected learning process script. The middle part is split into two sections; the upper one is used for the general settings of the learning process, while the lower one is intended for user management. The general settings are those properties related to the PBL module as a whole, while the user management part helps in actor management by assigning real registered users to the actor organization model of the learning process.

A learning process can have different general settings and different participants. On the right-side panel, the real registered users are listed. Users can be added to or removed from the organization model. Their user information will be then added to or removed from the specific actor in the lower panel of the middle part. Once a user is added to the list of the participants in a particular actor, his or her context-appropriate role can be decided. For instance, a learner-participant can have the role of an *ordinary learner* or a *group leader*, while a teacher-participant can be assigned a role such as a *facilitator* or *supervisor*.

As shown in Fig. 5, the designer has created two instances of PBL script modules and “Instance 1 of the deformed frogs” is selected. All the corresponding actors facilitator, class, group 1, and group 2 are then listed in the actor panel below. In this panel, group 2 is selected and all the currently enrolled participants are shown right next to it. In the organization model, group 2 is set to have four to five participants. Once the number of the participants reaches to four and is not more than five, group 2 will become in a “valid” state. The PBL module is in a “ready-to-run” state if and only if all the actors are in the valid state. The tool will automatically deliver the module to a module repository after the module is ready-to-run. The state of the module will be changed to “running” at the moment the involved participants start performing the learning process in the run-time environment which will be described in the next sub-section.

### The PBL-specific run-time environment

It has been shown that PBL processes can be designed using the PBL authoring tool and instantiated through the instantiation tool. As a result, the abstract processes become runnable. In this section, we will show that a run-time environment has been specifically developed to handle the runnable PBL modules in order to carry out the learning activities for learning participants. The run-time environment has been developed in response to the fact that the existing learning script players are not designed with PBL in mind. Consequently, they lack support of the elements that are essential in the PBL pedagogy.

**Fig. 6:**
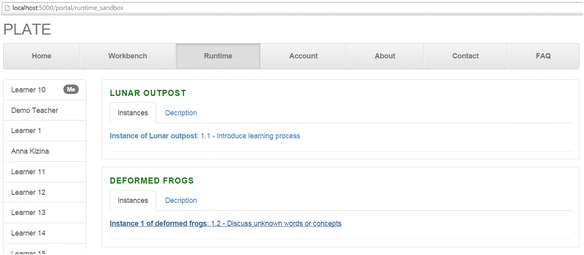
The user agent of the native run-time environment

**Fig. 7:**
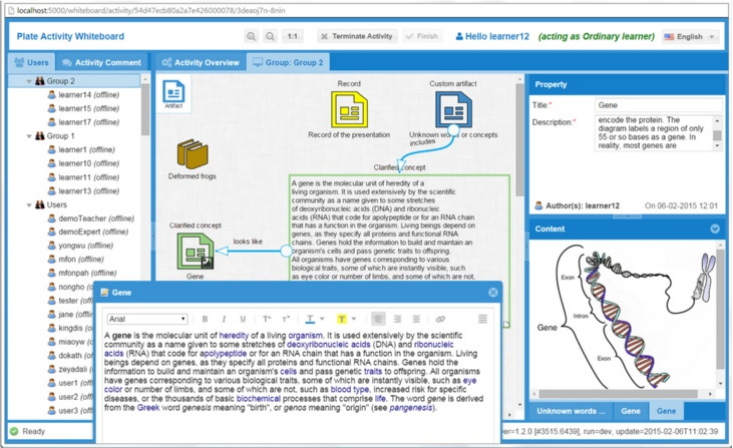
The UI of the PBL-specific whiteboard

Our run-time environment makes use of a *user agent* shown in Fig. 6 and a PBL-specific *whiteboard* depicted in Fig. 7. The user agent is responsible for publishing and updating the status information of PBL modules for different participants according to the arrangements. Through the user agent, facilitators and learners are encountered to only those learning modules and activities that they are involved in. Facilitators, however, have higher privileges such as monitoring the activities and the progress made by the learners, which makes it easier for them to actively support learners in their learning activity.

All the planned learning activities are collaboratively performed by participants in the PBL-specific whiteboard. The term “whiteboard” is often used in PBL community as three-column or four-column whiteboard at the front of the classroom. In this paper, we extend this concept as a shared run-time learning space that can be used by individuals in blended or distance PBL session. The whiteboard assists learners and/or facilitators to carry out their collaborative learning task activity by activity according to the learning process definition. When the whiteboard is displayed, the current learning activity-associated learning resources, learning tools, and expected final artifacts are already put there.

At the left of the whiteboard, a panel shows all participants according to the actor organization, current activity, and the instantiation arrangement. This panel makes the participants aware of their group members and also other groups. It also shows their status whether online or offline. Chatting session can be established between two participants, or within a group, or for a particular learning topic which is bound with a particular learning artifact. A learning space is located on the right side of the whiteboard. This space provides a shared place for participants who are in the same group. It could also become a private space if a participant is set to individually perform the current learning activity. Similar to the authoring tool, participants can create or edit artifacts on the center learning space to represent their intermediate or final learning outcome.

Continue the example, as shown in Fig. 6, “learner 10” can start activity 1.2 “Discuss unknown words or concepts” of the module “Instance 1 of the deformed frogs” from the user agent. Once a participant clicks the link to start this activity, the PBL-specific whiteboard, shown in Fig. 7, will be opened to carry out this activity for the learning process. In the whiteboard, this participant will view the given learning resources by clicking the deformed frogs icon. And he or she will freely contribute his or her ideas or knowledge artifacts via adding texts or links; uploading images, audios or videos; embedding external web sites; etc. Definitely, this freedom is also controlled by the whiteboard according to the role set to the participant. For example, if the participant is an ordinary learner, he or she can only update his own artifacts; if the participant is a group leader, he or she is able to update not only his own artifacts but also the group members’ artifacts. Usually, the facilitator has the right to terminate an activity. Once this activity is finished by participants or terminated by their facilitator, the content of the whiteboard will be updated for the next activity. For more details about the collaborative operation control, another dedicated paper will be issued.

## A model-driven approach

The previous section has shown an implicit but very important fact regarding the whole application: Every next step of technology-enhanced support is based on the previous result. Thus, a PBL process gradually becomes a concrete runnable learning module originating from the teacher’s initial ideas. In fact, this kind of evolution process is a model transformation process because the models, such as the actor organization diagram, the PBL process diagram, and runnable PBL module, play an essential role in representing or producing PBL domain knowledge as well as ensure the representation or production being manipulable by the computer.

In order to systematically support this multi-step model transformation, we adopt a model-driven approach. Consequently, we analyzed, designed, and developed this application under the methodology of model-driven engineering (MDE) (Kent 2002; Schmidt 2006). MDE is a software engineering methodology which combines process and analysis with model-driven architecture (MDA). In MDE, MDA approach plays a core role. From the illustration of the previous section, after analysis, we can find that the MDA fits very well to our application requirements. This match is presented in several ways as follows according to OMG (Object Management Group)’s MDA guide (OMG 2003; OMG 2014):

- The MDA approach provides a complete set of support from requirements to modeling to technology implementation. This gives a systematical support for the development of the PBL application in the context of our application requirements.
- MDA models can be used for the production of technology artifacts and executable systems. In the context of our application, the technology artifacts are the PBL process scripts; the executable systems match to our PBL-specific run-time environment and the IMS-LD-compatible players.
- The MDA is better to deal with interaction between organizations, people, software, etc. For example, teachers’ designs are able to be shared from experts to teachers to students through the graphical representations or the auto-generated textual description; besides, the PBL process scripts are able to run in the IMS-LD-compatible players after a transformation basing on the IMS-LD specification.
- The MDA approach is good for the application life cycle evolution since people can provide better executable systems by just improving the underlying models. As we mentioned before, to achieve a full power, an online or blended PBL process may need years of research, application, assessment, and redesign. However, compared to the refactoring of the hard-coded PBL applications by professional developers, this approach makes PBL designers able to improve the computer-supported PBL only by improving the PBL process model.

For applying the model-driven approach, an important premise is to be clear about the target application domain. Consequently, a domain-specific modeling language (DSML) must be defined. Obviously, the application domain of our case is the PBL domain, and therefore, we need a PBL domain-specific modeling language (PBL-DSML). According to Schmidt (2006), we can make a PBL-DSML to define the relationships among concepts in the PBL domain and precisely specify the key semantics and constraints associated with these domain concepts. As a result, PBL designers are able to build their PBL process models using elements of the type system provided by the modeling language, and are guided and constrained to express their process design declaratively rather than imperatively, which will make the process design work become much easier.

Although OMG’s MDA approach suggests to use UML as a basis and although there are also other modeling languages such as Business Process Model and Notation (BPMN), we believe that it is better to derive the PBL-DSML concept directly from computer-supported collaborative learning (CSCL) scripts. Indeed, computer-supported PBL should be conceived as a kind of CSCL and thus a CSCL scripting language would be a kind of DSML. As we know, CSCL scripts have been considered an effective means of facilitating specific interaction patterns in CSCL situations (Fischer et al. 2007). And numerous approaches to representing CSCL scripts and CSCL scripting languages have been reported in the literature (e.g., Dillenbourg 2002; Miao et al. 2007; Dillenbourg and Tchounikine 2007; Harrer et al. 2007). Nonetheless, these CSCL scripting languages provide inadequate support for PBL, since the CSCL is yet for modeling the general learning activity. Thus, based on the existing CSCL scripting languages, combined with the domain concepts of the PBL as well as emphasizing supporting the particular PBL pedagogy, a PBL scripting language has been proposed (Miao et al. 2007; Wang et al. 2014). This language is developed after analyzing the features of all existing mainstream PBL models in case of supporting a general expressiveness for representing various forms of PBL processes. Notice that the scripting language is also referred to as learning process meta-model (Devedzić 2002; Atkinson and Kuhne 2003; Aßmann et al. 2006) under our application context. The meta-model is also configurable. This will be discussed in more detail in our other papers.

Since the vocabularies and rules of the PBL scripting language are specified by PBL domain experts, rather than using generic vocabularies, this language uses concepts that teachers use daily to describe PBL processes. For instance, as shown in the previous section, in the language, the learning phases include *problem engagement*, *identify learning issue*, *generate solutions*, *evaluate acquired knowledge*, etc.; the activities under the learning phase problem engagement include *meet problem*, *introduce problem*, *clarify concept/term*, etc.; the element concepts include *activity*, *resource*, *tool*, *actor*, *artifact*, etc.; and the actor element could refer to a *facilitator*, *individual*, or *group*. As mentioned above, the abstraction of these concepts is at the same level of the daily used terms of PBL teachers. Therefore, teachers do not need to concern about the specific technical requirements and the correctness of the syntax and semantic when designing computer-supported PBL processes but mainly only need to focus on the element property configuration and element relationship declaration.

**Fig. 8:**
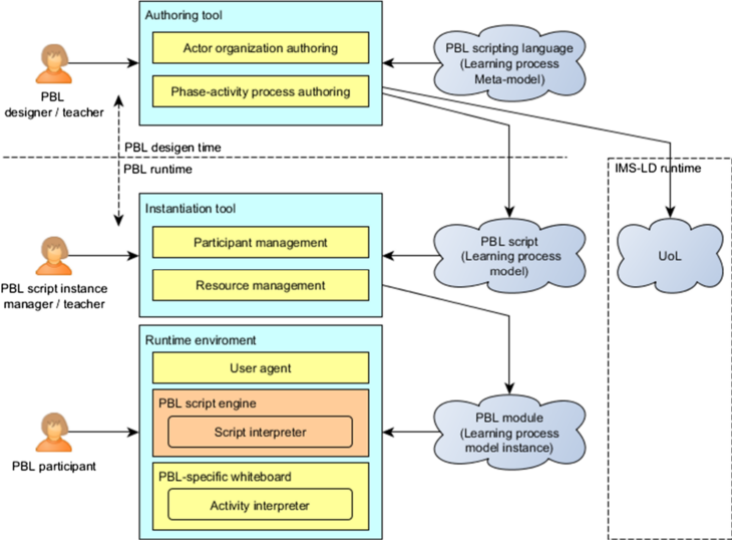
The model-driven architecture inside the PBL application

Figure 8 illustrates the underlying MDA of our PBL application. This diagram shows that the system is designed to have two parts: the PBL design time and the PBL run time. The PBL authoring tool belongs to the design time; the instantiation tool and the run-time environment belongs to the run time. In the design time, the authoring tool is driven by the PBL scripting language (learning process meta-model). Supported by the authoring tool, the PBL designer can design the PBL process by authoring actor organization and the phase-activity processes. As a result, a PBL script (learning process model) will be produced. Since the PBL scripting language is designed concerning the interoperability with IMS-LD, the PBL script is able to be transformed to a unit of learning (UoL) and run in IMS-LD-compatible run-time players.

In the run time, the designer first uses the instantiation tool to instantiate a PBL process script into a PBL module through arranging participants (and learning resources). The process is then delivered as a PBL module (learning process model instance) which is executable in the run-time environment. In the PBL-specific run-time environment, a user agent manages the status of modules and publishes them to involved participants; a PBL script engine is responsible for interpreting the module based on the syntaxes and the semantics defined in the scripting language. The engine interprets the learning process inside the module, activity by activity to assemble all information, such as the user information of participants, learning resources, learning tools, and excepted learning artifacts, for the current learning activity, and produces the information package as a module activity instance. The PBL-specific whiteboard interprets the activity instance to display all the learning activity information and handle all the expected learning interaction for learning participants.

**Fig. 9:**
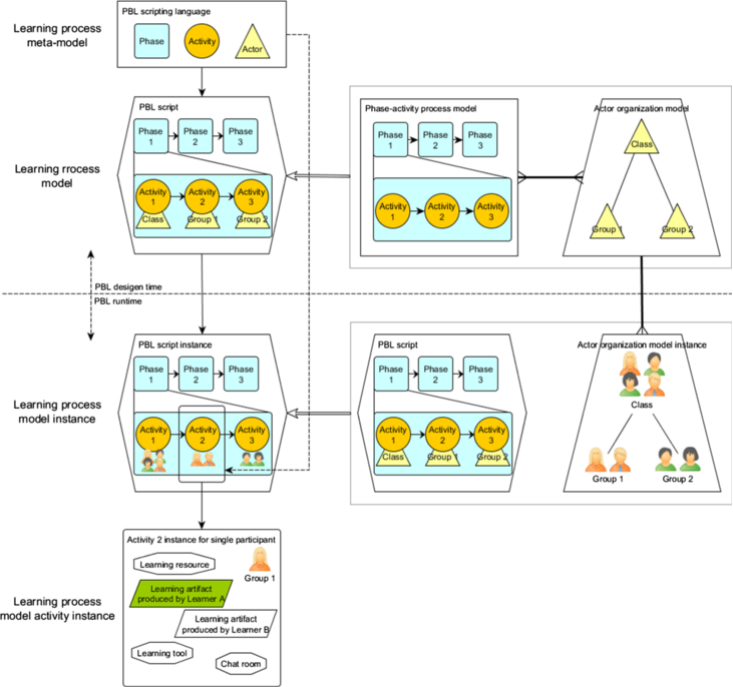
The model transformation in the model-driven architecture

Figure 9 illustrates our model-driven architecture from the perspective of model transformation (for simplifying the presentation, this figure does not involve the instantiation of learning resources, etc.). In this application, the PBL scripting language, as a domain-specific modeling language, is the highest level model in the model-driven architecture driving the whole system. As mentioned above, the scripting language is the meta-model of a learning process model. In the design time, the *phase*, *activity*, *actor*, etc. are the meta-elements—from the type system of the meta-model. These meta-elements are presented as the building blocks to designers for presenting the PBL processes. The authoring operations of designers make the single learning process meta-model (PBL scripting language) transform to learning process models (PBL scripts). A PBL learning process model consists of a phase-activity process model and an actor organization model. The relationship between these two models is many-to-many, which means a phase-activity process can be alternatively combined to different actor organization models and vice versa.

At run time, a learning process model (PBL script) is transformed to learning process model instances (runnable scripts) by arranging the different groups of real system users into the actor organization model (and by setting different learning resources, configuring different session start time, choosing different target run-time environments). The relationship between the organization model and the participant arrangement is one-to-many, which means an organization model is able to have different arrangements. This enables a PBL script to have infinite forms of instances. Finally, according to the specification of the learning process meta-model (the PBL scripting language), the possibility of interpreting every detailed interactive information of each activity in the learning process model instance is guaranteed.

It is important that, according to OMG’s MDA, the PBL script belongs to the platform-independent model (PIM) and the PBL script instance belongs to the platform-specific model (PSM). Actually, there are already a number of tools which support the PIM to PSM transformation, such as some computer-aided software engineering (CASE) tools. Nonetheless, they are mostly used for the code generation and designed for the technical people in the domain of software engineering such as software developers but not for the non-technical people such as PBL practitioners.

## Supporting the manipulation of PBL processes under the model-driven approach

The previous section explains the way to meet the requirements of computer-supported PBL from the modeling point of view. \However, before actually implementing it, there are still several technical difficulties which need to be overcome in order to implement the MDA, for example, (1) how to represent and handle the networked graphical actor organization and the multi-level structured learning process in the design time especially when the depth and the degree of the representation are uncertain; (2) how to effectively operate the model elements where the operation includes save, retrieve, interpret or transform all the elements, all the properties of every element, all the relationships between the elements, etc. especially when the elements are constantly changed with different levels of model abstraction; (3) how to transform a multi-level graphical process to a complete process script or a textual document; and (4) how to interpret the script to a PBL module for the run-time environment. Actually, all these difficulties are caused by the fact that the structure of the process models and the element information inside the models in our application are semi-structured.

### Handling semi-structured process models

According to Buneman (1997), for the semi-structured data, either there is no separate schema to constrain the information or the schema exists but only implies loose constraints. Under this description, the PBL process models are semi-structured, since there is no schema to constrain the depth and degree of the processes. Although the models are semi-structured and very flexible, they still have a homogeneous hierarchical characteristic: every next level in a model is the definition of an element in the previous level; and within the same level, the information is only about the properties of the elements and the relationship between the elements. Basing on this characteristic, therefore, the authoring workspace can be also designed to support the representation of the PBL processes hierarchically, which means each edit space (we call it workspace in the “Characterize PBL and identify technical requirements” section from the user’s viewpoint) represents the definition of the upper layer element. The definition of each layer is a directed graph that consists of elements and connections.

**Fig. 10:**
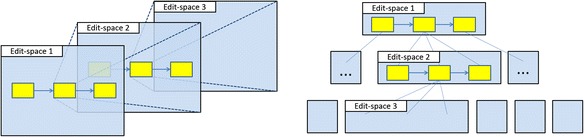
The hierarchical representation concept of a PBL process in multi-level edit spaces

Figure 10 illustrates this kind of hierarchical process representation from two aspects. Edit space 1 is for designing the phase sequence, for example. After a phase was defined, it can be opened into a new edit space. Similar to the first level, in edit space 2, activities, resources, tools, actors, artifacts, and their relationships can be defined. If required, a third level of edit space can be opened. Therefore, a process script even with infinite depth or degree is possible to be defined and represented. Since authoring operation requirements in each level are similar, the functionalities required for each edit space are similar too.

Correspondingly, the database can be also designed to save or retrieve the process elements and the relationship iteratively like the representation manner in order to handle the infinite depth and degree of the script data. Because there are only finite meta-element types (phase, activity, resource, tool, actor, and artifact) in the meta-model (the PBL scripting language) basing on the design of our MDA, all elements can be saved or retrieved in their own meta-element types of collections. Relationships among elements are seen as connection element which can be saved or retrieved in a connection collection.

**Fig. 11:**
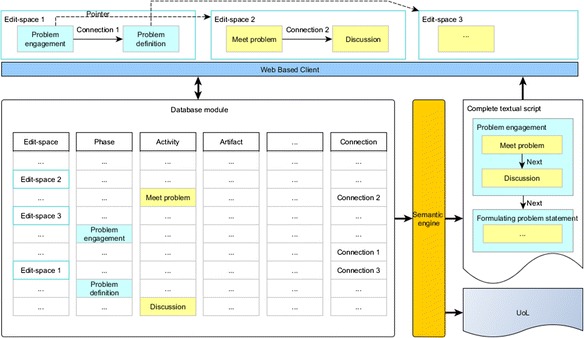
The storing and retrieving concept of PBL scripts

Figure 11 illustrates this kind of data saving and retrieving approach. Specifically, each element has a *definition id* that points at another edit space; each edit space has a *segment id* to let the elements and connections know which edit space they belong to. Elements and connections of different edit spaces from different scripts are stored together in their own meta-element types of collections. The retrieving of a definition is to find all the elements and connections where their segment id equals to the edit space’s segment id. Then, the data can be directly sent back without any process logical calculation. Based on their graphic coordinate information, all elements and connections will be correctly put back on the corresponding edit space. When the designer wants to view the complete script, all elements and connections can be easily retrieved because all of them who belong to one script have the same *script id*. The only additional thing is that a semantic engine will assemble them into a correct order. Also because of this engine, the authoring tool is able to produce the script as a UoL for IMS-LD-compatible players as well.

Because the elements and the connections are stored separately, it shows several benefits: (1) It is highly efficient to add, remove, update, and find one element wherever it is in one or multiple PBL scripts. When a designer edits an element, changes can be updated to the target element without searching all the elements of all models. This benefit is effective for reducing the computation cost in semi-structured data searching. (2) It is very easy to handle the change of the process structure. Because the elements are not nested to each other, the change of structure only affects the change of the definition id and the connection element. This benefit makes it possible to effectively update, reuse, and share any level of sub-process among the PBL process scripts.

### Handling semi-structured element data in the process models

The previous section has elaborated the approach to handle learning processes. However, not only the structure of the PBL process is semi-structured but also the element information itself is semi-structured.

**Fig. 12:**
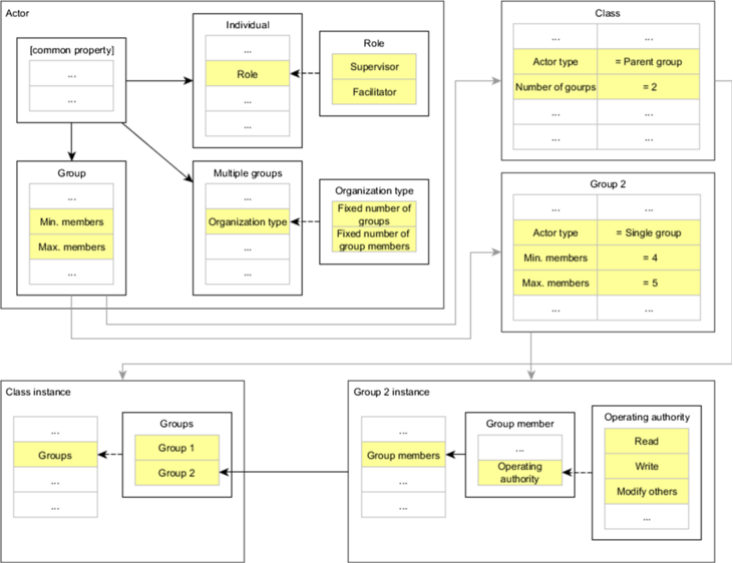
Constantly changed properties of model elements in different levels of models

As illustrated, our system applies MDA, and the elements of models will be constantly changed in order to adapt different levels of process abstraction during the model transformation. Therefore, from the process element point of view, as an example, the change of information inside elements could be shown in Fig. 12. In this diagram, we use the actor element to show how the properties tend to be constantly changing in different modeling stages. When the actor element belongs to the meta-model, the application needs to store all the property definition (and type definition, relationship definition) and the corresponding option values. For example, according to the scripting language, the meta-element *multiple groups* has a single choice property named *organization type*, and it has the options of *fixed number of groups* and *fixed number of group members*. But for the meta-element *group*, it does not have this property. However it has other properties such as *min. members* and *max. members*. Furthermore, although a *class* element or a *group 2* element is created basing on the same meta-element *group*, the property fields are different. According to the storing strategy illustrated before, these two elements need to be saved in the same data collection: the *actor* collection. Also because of this difference, when the *class* and *group 2* are instantiated, the instance results are consequently quite different with each other. As shown in the figure, *class instance* need to save the grouping information, while the *group 2 instance* only concerns the group members’ information and their operation policy. In summary, the number, depth, and types of the properties need to be stored in a very flexible manner.

As a web-based application, also considering to avoid browser compatibility issues and to realize the semi-structured flexibility, we have chosen Node.js as our web server and JavaScript and JSON to build both client-side and server-side modules. Also, we use a NoSQL database to handle the semi-structured data storage and retrieval. The reason why this combination of technologies is effective to handle the semi-structured element data is that JSON is an ideal data-interchange language for representing semi-structured data and it is a subset of the object literal notation of JavaScript. The element and connection objects in the application could simply be JSON objects. Consequently, on the one hand, client side and server side can communicate with each other directly through a JSON object (after serialization), which means, in the client side, elements and connections can be directly rendered to edit space or sent back to the server side without any format transformation. Also without any transformation, server side can directly process the elements or connections from the client side. On the other hand, data from the client side can be stored inside the database directly, even if there are different numbers, depths, and types of the properties in the same type of object. For example, the client- and server-side JavaScript interpreter and the MongoDB, a NoSQL technique, can handle and store the element class and group 2 as a JSON object natively.

**Fig. 13:**
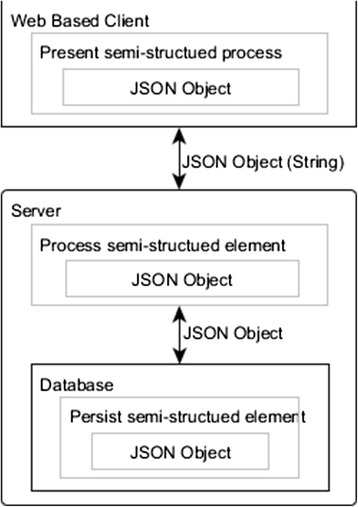
System architecture from the perspective of the technical composition

Figure 13 shows the system architecture from the perspective of handling the semi-structured element information. Driven by the PBL scripting language, together with using pure JavaScript for the client UI, Node.js as the web server, MongoDB for the data persistence, JSON as the unified semi-structured data format in the whole system, and with the approach for handling the semi-structured process models, all the technical requirements for implementing an application that is able to support authoring, delivering, and execution of PBL processes are met.

## Related work

Currently, there are two kinds of implementations that can flexibly support the PBL design: IMS-LD authoring tools and the LAMS (Dalziel 2003). About IMS-LD authoring tools, there are Reload (Reload 2005), MOT+ (Paquette et al. 2006), ASK-LDT (Karampiperis and Sampson 2005), CopperAuthor (CopperAuthor 2005), and CoSMoS (Miao 2005). These tools are very flexible to represent and support the design of different learning process models which include PBL models, and they belong to general learning design tools. About the LAMS, a study has shown that it also can be used for the PBL design (Richards and Cameron 2008).

However, all of those tools above have some shortcomings in terms of supporting the specificities of the PBL design and implementation. From the perspective of supporting visual learning design, IMS-LD authoring tools and the LAMS miss the capability of facilitating teachers in developing a sound PBL process since they are too general and not PBL domain specific. Using IMS-LD authoring tools or the LAMS, users have to explicitly represent PBL features using higher abstract building blocks and data types. For example, they neither have the building blocks such as PBL-specific activity or artifact nor provide the types such as problem engagement or identify learning issue which are emphasized in PBL pedagogy. From the perspective of utilizing web technologies, some of these tools came about by adopting traditional software development concept, and most of them are desktop applications. As we know, desktop applications have high maintenance cost, are not anywhere available without pre-installation, and so further. From the perspective of data management approach, existing applications store learning design artifacts by using either traditional relational database or directly as XML files. Although relational databases are good at data storage and querying, they cannot manage this kind of semi-structured data well and flexible enough since they are relational and not schema free. XML file is a kind of ideal media for storing this kind of semi-structured data; one drawback is that it is not ideal for the data manipulation such as partial update, search, and sub-document sharing. Although there are some combination solutions to manage XML documents through relational database, XML queries are still inefficient (Shanmugasundaram et al. 2008).

For supporting the PBL implementation, people possibly use the IMS-LD authoring tools to design PBL processes and produce the UoLs. Then, the UoLs can be interpreted by IMS-LD run-time players, such as CopperCore player (Martens and Vogten 2005) and SLED (McAndrew et al. 2005) in order to help the PBL implementation. Our application aims to be able generate the UoLs under the IMS-LD specification, so that we can make use of these existing run-time tools.

## Summary and future work

PBL is a rich and highly collaborative learning process with a specific pedagogy. Therefore, designing and implementing a PBL process is not an easy task. The difficulties include specifying actor roles, designing a process with phases and activities, and instantiating and enacting an adequate process script. In order to represent this kind of learning process, based on the concepts learned from CSCL scripting languages, our PBL scripting language has been developed. Applying this language in a Web 2.0 context, we developed a web-based PBL application. This application provides a set of rich and intuitive authoring, delivery, and execution tools for helping teacher to design and implement PBL processes applying different PBL models.

We extracted several different abstract levels of models in order to support the different steps of requirements for supporting technology-enhanced PBL. These different level of models include a PBL process meta-model (PBL scripting language), a PBL process model (PBL process script), and a PBL process model instance (PBL module). This extraction is fit well for applying the MDA approach. Along with several other important benefits from this approach, therefore, we design and develop this application under the methodology of MDE. This application is implemented based on the premise that there are two dimensions of semi-structured characteristics inside PBL processes.

Our former pilot studies including Miao et al. (2013, 2014, 2015) have shown that the difficulties to design and deliver a pedagogically high-quality, human-readable, reusable, sharable, and computer-executable online or blended PBL process are reduced as intended. For instance, a pilot study (Miao et al. 2015) was conducted at the College of Education in Qatar University. The participants were students from a Masters in Education program, and they were about to end an advanced curriculum development and design course. Most of the participants are meanwhile working as teachers in primary, preparatory, and secondary schools or not as teachers but working in the education-relevant fields. In the pilot study, we asked several open-ended questions in relation to the usefulness of the tool in designing an online PBL process. Feedback from the participants includes “I used the PBL Workbench for a science lesson. It was suitable for the topic.,” “When I used the PBL Workbench I did not have any difficulties performing a task. There were various possibilities to work with.,” “I liked the way. It allows connections to be made between various elements, actors, activities, etc. I also liked that it provides clarity to every phase and activity as it asks for goals and other details.,” “This tool was amazing in helping me develop the plan of how to conduct performance management at the school especially with the complications of connections to be made.,” and “It was new and exciting experience for me.” This is just one example feedback of many to show the result of our quantitative evaluations of the application.

This paper systematically elaborates an approach to design and implement a web-based PBL authoring and run-time application to help teachers to achieve their PBL practice goal. Both from technical viewpoints and from qualitative viewpoints, we conclude that a combined use of the model-driven approach and semi-structure-oriented data management appears to be a promising approach to effectively and efficiently support the authoring, delivering, and execution of PBL processes. The future work could have several directions: the one could be to make the script transformation steps automatically. That is, extract process directly from existing semi-structured textural scripts and then store them in the system, represent them by our graphical user interface, transform them to other learning systems, or directly run them; or to research and develop a kind of new search engine in order to retrieve similar processes based a target scripts—a pattern based search; or to conduct more evaluations to the authoring tool and the run-time environment, so that we can further improve either the PBL application or the PBL scripting language.
